# 

**Published:** 1916-03

**Authors:** 


### CONTENTS.

ORIGINAL COMMUNICATIONS—

Strangulated Hernia With Operation Described and Illustrated. By L. C. Fisher, M. D., Atlanta, Ga. ........ 569

Causes and Types of Acidosis. By Edgar G. Ballenger, M. D., F. A. C. S. and Omar F. Elder, M. D., Atlanta, Ga. ........ 583

Acetonemia and Vomiting in Children. By Howard Bucknell, M. D. ........ 586

Establishment and Work of a Free Clinic for Children in Montgomery, Ala. By G. J. Griel, M. D., Montgomery, Ala. ........ 596

A Clinical Paradox. By George M. Niles, M. D. Atlanta, Ga. ........ 599

EDITORIAL ........ 601

SELECTIONS AND ABSTRACTS ........ 609

